# Supplementary material for: NogoA-expressing astrocytes limit peripheral macrophage infiltration after ischemic brain injury in primates
Source: Nat Commun. 2021 Nov 25;12:6906. doi: 10.1038/s41467-021-27245-0 (PMC8617297; doi:10.1038/s41467-021-27245-0)
Supplement: Supplementary file 1 — Supplementary Information [file 41467_2021_27245_MOESM1_ESM.pdf]

Supplementary Information:

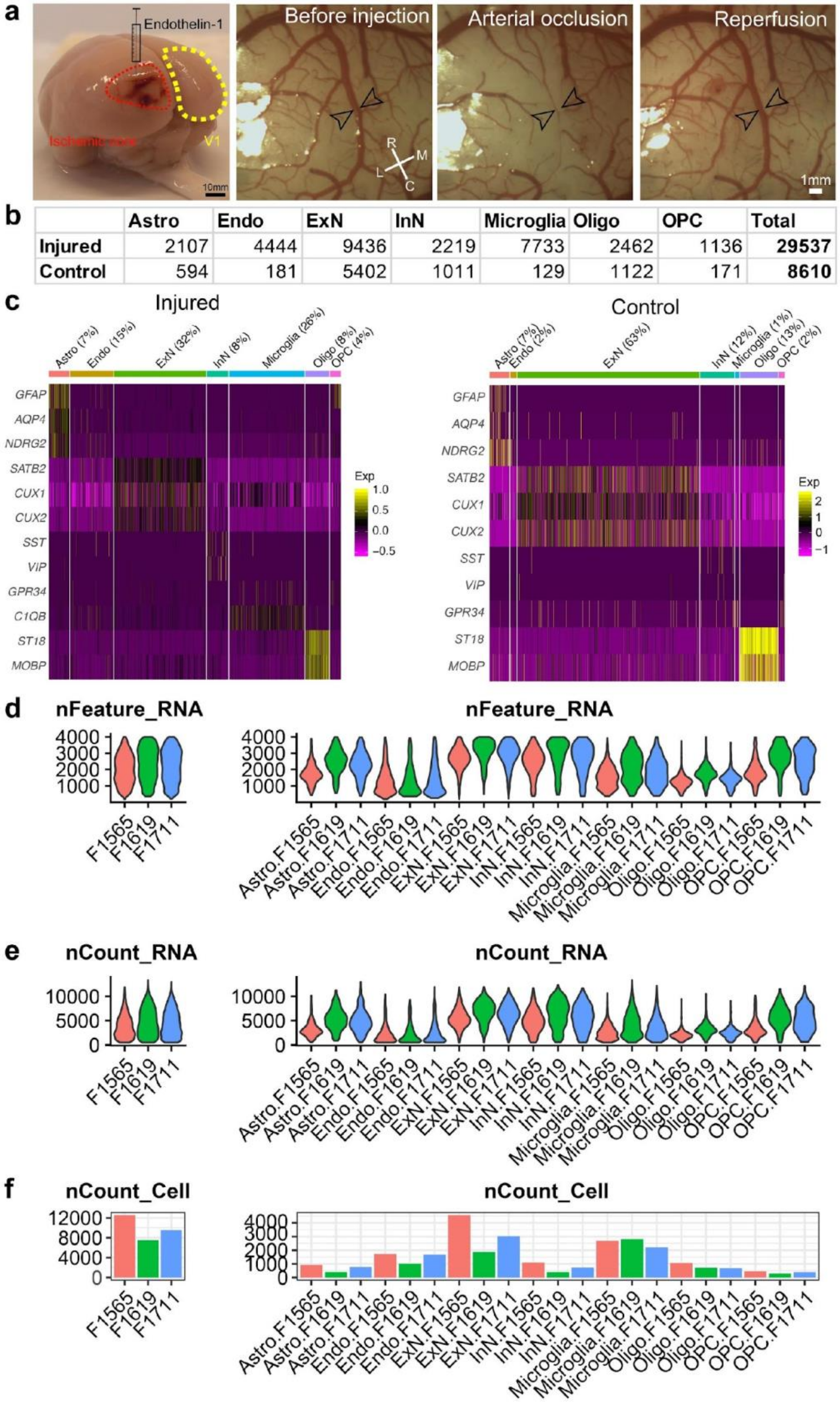

Supplementary Fig. 1. Marmoset ischemic stroke induction with vasoconstrictor and astrocyte identification for transcriptomics. (a) Far left; one-week post-endothelin-1-induced ischemia of marmoset caudal neocortex (red hatched area ischemic core and yellow hatched area V1 contralateral, used as uninjured control) and pre- (Middle left)/ post- (Middle & far right images) injection time course highlighting transient ischemia. Arrowheads: occlusion of the posterior cerebral artery. (b) Summary table of single nuclei numbers for each cell type in injured and control cohorts (c) Heat map colored by single nuclei gene expression of remaining cell-type specific markers; accompanies Fig. 1c. (d) Violin plots showing the number of genes detected in each individual injured marmoset (left), and in each cell type from each individual injured marmoset (right). (e) Violin plots showing the distribution of the number of unique molecular identifiers (UMIs) detected in each individual injured marmoset (left), and in each cell type from each individual injured marmoset (right). (f) Bar graphs showing the distribution of the number of cells captured in each individual injured marmoset (left), and in each cell type from each individual injured marmoset (right). Astro: astrocyte; Endo: endothelial cell; ExN: excitatory neurons; InN: inhibitory neurons; Oligo: oligodendrocytes; OPC: oligodendrocyte precursor cells.

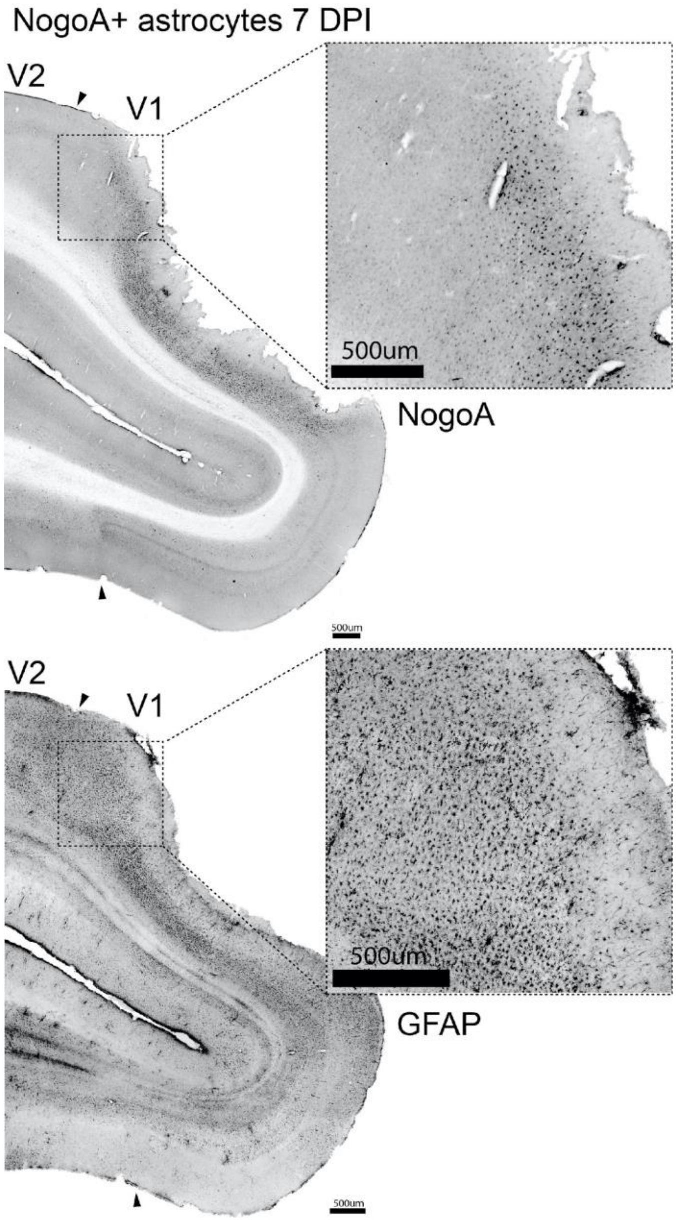
NogoA+ astrocytes 7 DPI
V2
V1
500um
NogoA
500um
V2
V1
500um
GFAP
500um

24

Supplementary Fig. 2. Sagittal view of NogoA and GFAP expression at the V1/ V2 border post-ischemic stroke. Photomicrographs of marmoset sections 7 DPI (left) and magnified region of interest depicted by black hatched line (right). NogoA (top) and GFAP (bottom) were labelled by DAB immunohistochemistry (n=3 biological replicates). DPI: days post-ischemia; V2: secondary visual area; V1: primary visual cortex; NogoA: neurite outgrowth inhibitor A; GFAP: glial fibrillary acidic protein.

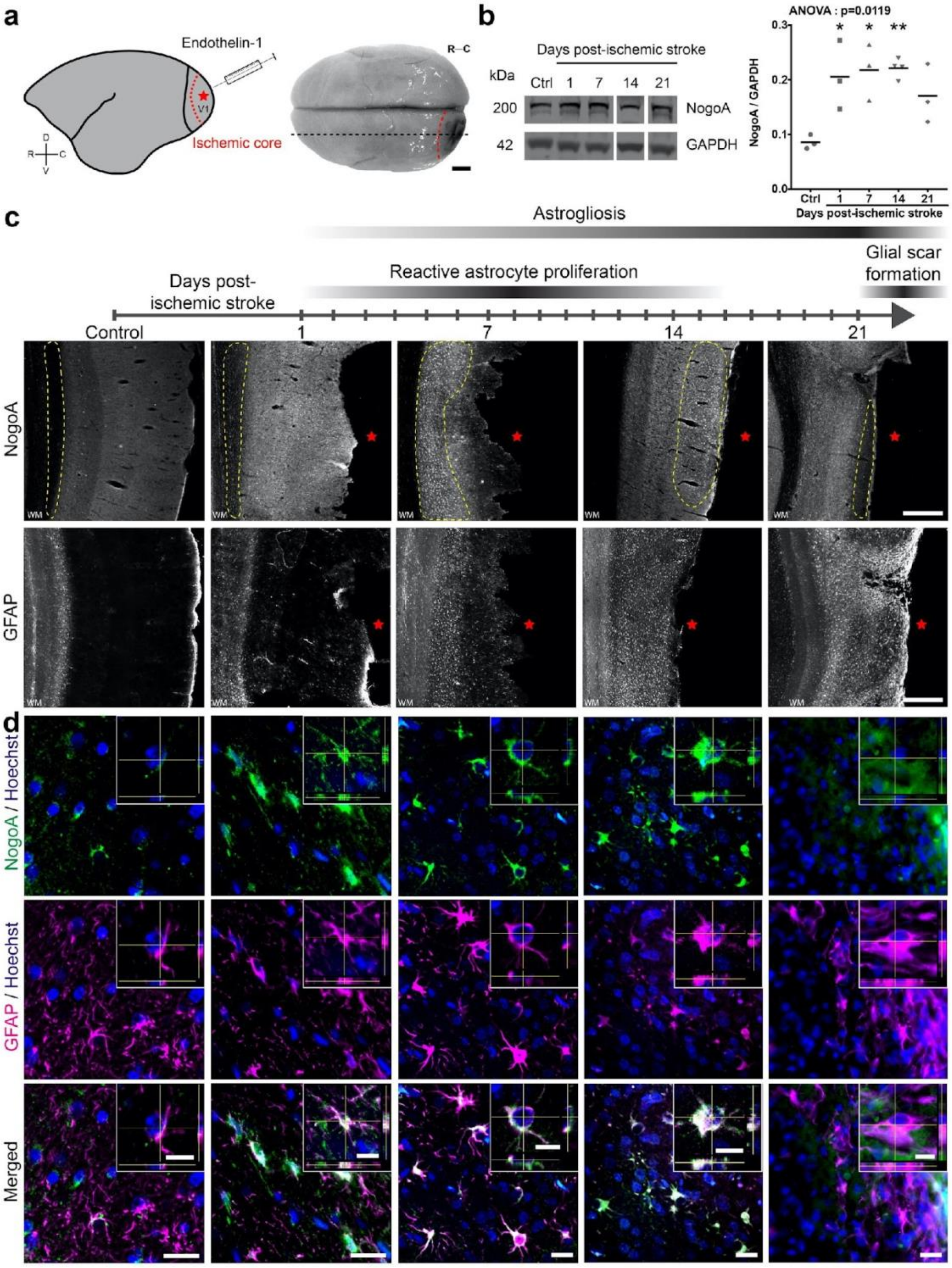
a
Endothelin-1
V1
Ischemic core
D
R
C
V
R–C
b
Days post-ischemic stroke
kDa
Ctrl
1
7
14
21
200
NogoA
42
GAPDH
ANOVA : p=0.0119
NogoA / GAPDH
0.3
0.2
0.1
0.0
Days post-ischemic stroke
c
Astrogliosis
Reactive astrocyte proliferation
Glial scar formation
Days post-ischemic stroke
Control
NogoA
GFAP
WM
d
NogoA / Hoechst
GFAP / Hoechst
Merged

Supplementary Fig. 3. NogoA is upregulated on marmoset GFAP+ astrocytes post-ischemic stroke. (a) Schematic and image of marmoset brain depicting ischemic-stroke induction, the region of analysis and lesion size relative to brain size. (b) Representative immunoblots for experimental time points analyzed and scatter plots depicting densitometric quantification of NogoA normalized to GAPDH. Each point represents the mean of 3-4 technical replicates for each marmoset biological replicate analyzed, n=3 per time point, excluding 14 DPI where n=4. (c) Schematic of astrocytic pathophysiological time course post-ischemic stroke in primates and representative images showing cellular profile of NogoA and GFAP within marmoset control V1 tissue and ischemic zones at 1, 7, 14 and 21 days post-ischemic stroke (DPI) by DAB immunohistochemistry (n=3 biological replicates/ cohort over several tissue sections comprising the ischemic core). (d) Representative confocal images and stacks with orthogonal views showing NogoA colocalization with GFAP+ cells within yellow dotted line region of interest from (c) in marmoset control V1 tissue and ischemic zones at 1, 7, 14 and 21 DPI (n=3 biological replicates/ cohort over several tissue sections comprising the ischemic core). V1: primary visual cortex; V2: secondary visual area; D: dorsal; V: ventral; R: rostral; C: caudal; kDa: kilodalton; NogoA: neurite outgrowth inhibitor A; GAPDH: glyceraldehyde 3-phosphate dehydrogenase; Ctrl: control; statistical test: ordinary one way-ANOVA with post-hoc Dunnett's multiple comparisons test; ANOVA p value=0.0119; *: $p<0.05$; **: $p<0.01$; WM: white matter; yellow dotted line: cell population of interest; red star: ischemic core; GFAP: glial fibrillary acidic protein; scale bars: 5mm (a), 500µm (c), 20µm (d: single images), 10µm (d: image stacks). Source data and tabulated statistics for (b) are provided as a Source Data file.

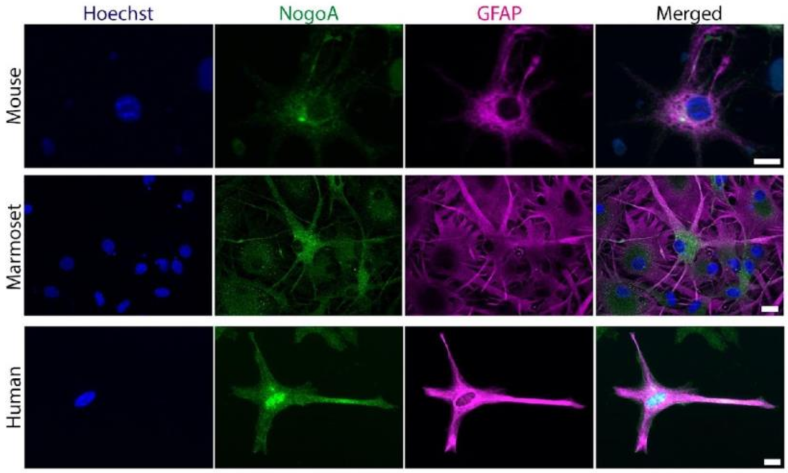
Hoechst
NogoA
GFAP
Merged
Mouse
Marmoset
Human

Supplementary Fig. 4. Mouse, marmoset and human astrocytes express NogoA *in vitro*, in the absence of myelin. **I**mmunofluorescent images showing expression of NogoA on GFAP+ mouse, marmoset and human astrocytes in culture (n=2 biological replicates/ species). GFAP: glial fibrillary acidic protein; NogoA: neurite outgrowth inhibitor A; scale bars: 20μm.

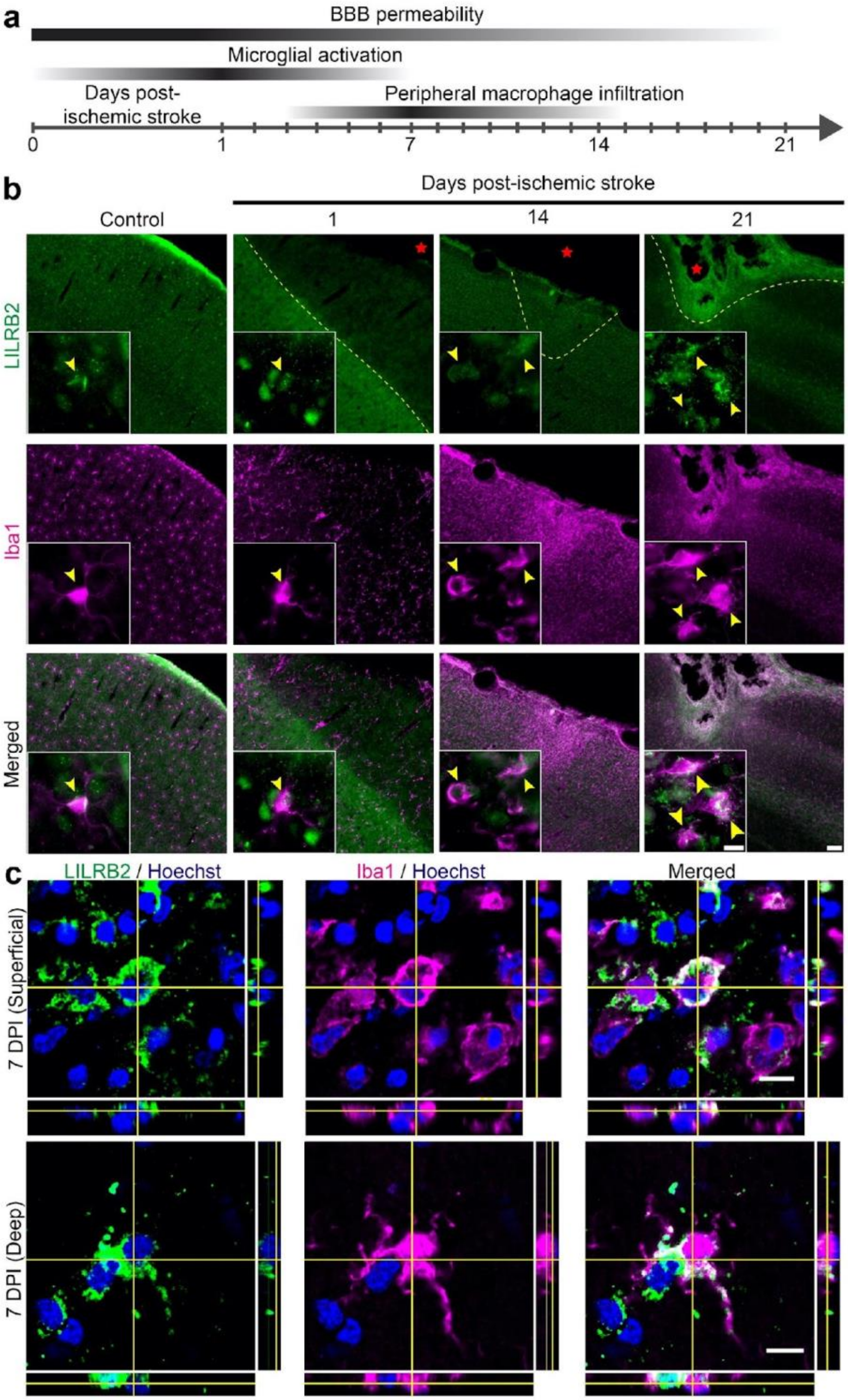
a
BBB permeability
Microglial activation
Days post-
ischemic stroke
Peripheral macrophage infiltration
0
1
7
14
21
b
Days post-ischemic stroke
Control
1
14
21
LILRB2
Iba1
Merged
c
LILRB2 / Hoechst
Iba1 / Hoechst
Merged
7 DPI (Superficial)
7 DPI (Deep)

Supplementary Fig. 5. LILRB2+/ Iba1+ macrophages at relevant post-ischemic stroke time points. (a) Schematic of macrophagic pathophysiological time course post-ischemic stroke in primates. (b) Representative immunofluorescent images showing LILRB2 colocalization with Iba1+ cells in marmoset control V1 tissue and ischemic zones at 1, 14 and 21 DPI. For 7 DPI time point refer to Fig. 4e. Magnified boxes depict double positive cells and morphologies identified within yellow dotted line region of interest in B (n=3 biological replicates/ cohort over several tissue sections comprising the ischemic core). (c) Confocal image stacks with orthogonal views showing LILRB2 colocalization with Iba1+ cells 7 DPI in superficial and deep cortical tissue (n=3 biological replicates/ cohort over several tissue sections comprising the ischemic core). LILRB2: leukocyte immunoglobulin-like receptor B2; Iba1: ionized calcium-binding adapter molecule 1; yellow dotted line: cell population of interest; red star: ischemic core; yellow arrowheads: LILRB2+/ Iba1+ macrophages; DPI: days post-ischemic stroke; scale bars: 100μm (b: larger box), 10μm (b: magnified box & c).

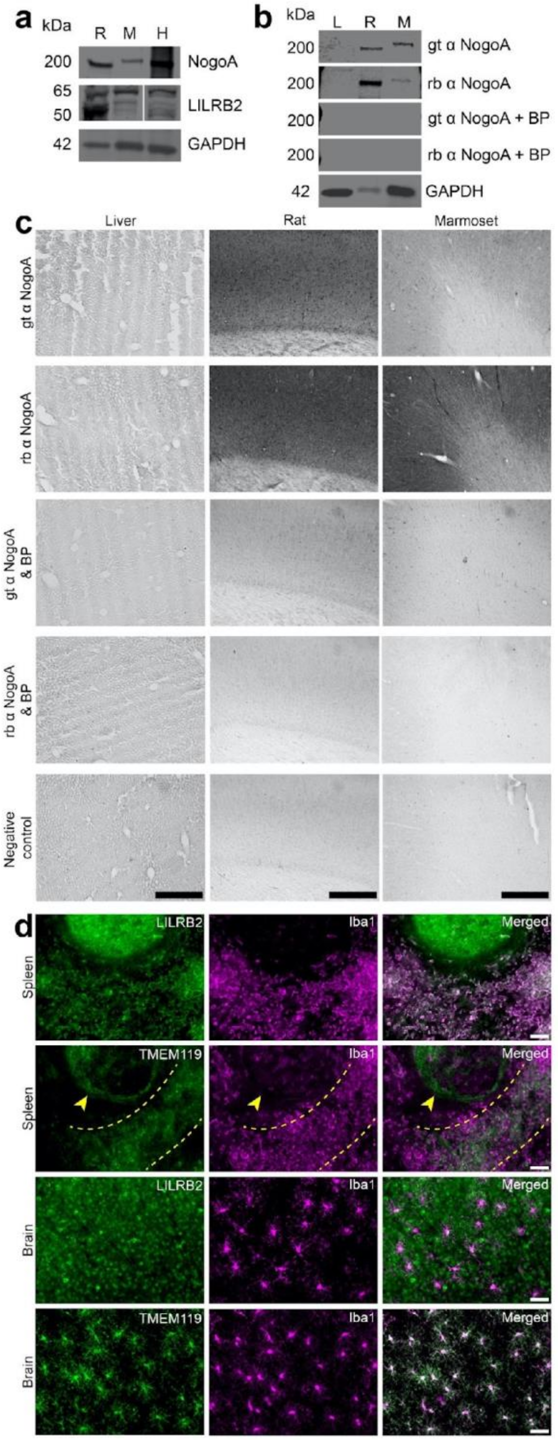
a
kDa
R
M
H
200
NogoA
65
50
LILRB2
42
GAPDH
b
kDa
L
R
M
200
gt α NogoA
200
rb α NogoA
200
gt α NogoA + BP
200
rb α NogoA + BP
42
GAPDH
c
Liver
Rat
Marmoset
gt α NogoA
rb α NogoA
gt α NogoA & BP
rb α NogoA & BP
Negative control
d
LILRB2
Iba1
Merged
Spleen
TMEM119
Brain

Supplementary Fig. 6. NogoA and LILRB2 antibody characterization in rat, marmoset and human. (a) Immunoblots of NogoA and LILRB2 in rat (lane 1), marmoset (lane 2) and human (lane 3) cortical tissue (n=2 biological replicates/ species repeated twice). (b) Immunoblots of two different NogoA antibodies in marmoset liver (lane 1), rat cortical tissue (lane 2) and marmoset cortical tissue (lane 3) (n=1 biological replicate/ tissue repeated twice). (c) Images show specificity of immunostaining by the two NogoA antibodies used in this study in marmoset liver, rat cortical tissue and marmoset cortical tissue (liver: n=1 biological replicate; rat cortical tissue: n=2 biological replicates; marmoset cortical tissue: n>3, over at least 3 tissue sections). (d) Images showing LILRB2 and TMEM119 co-labeled with Iba1 in marmoset spleen and brain (spleen: n=1 biological replicate; brain: n>3 biological replicates, over at least 3 tissue sections). NogoA: neurite outgrowth inhibitor A; LILRB2: leukocyte immunoglobulin-like receptor B2; GAPDH: glyceraldehyde 3-phosphate dehydrogenase; kDa: kilodalton; gt: goat; rb: rabbit; α: anti-; BP: blocking peptide; TMEM119: transmembrane protein 119; Iba1: ionized calcium-binding adapter molecule 1; yellow dotted line: reticular fibers of splenic cord; yellow arrowhead: lymph node capsule; scale bars: 500μm (c), 50μm (d). Source data are provided as a Source Data file.

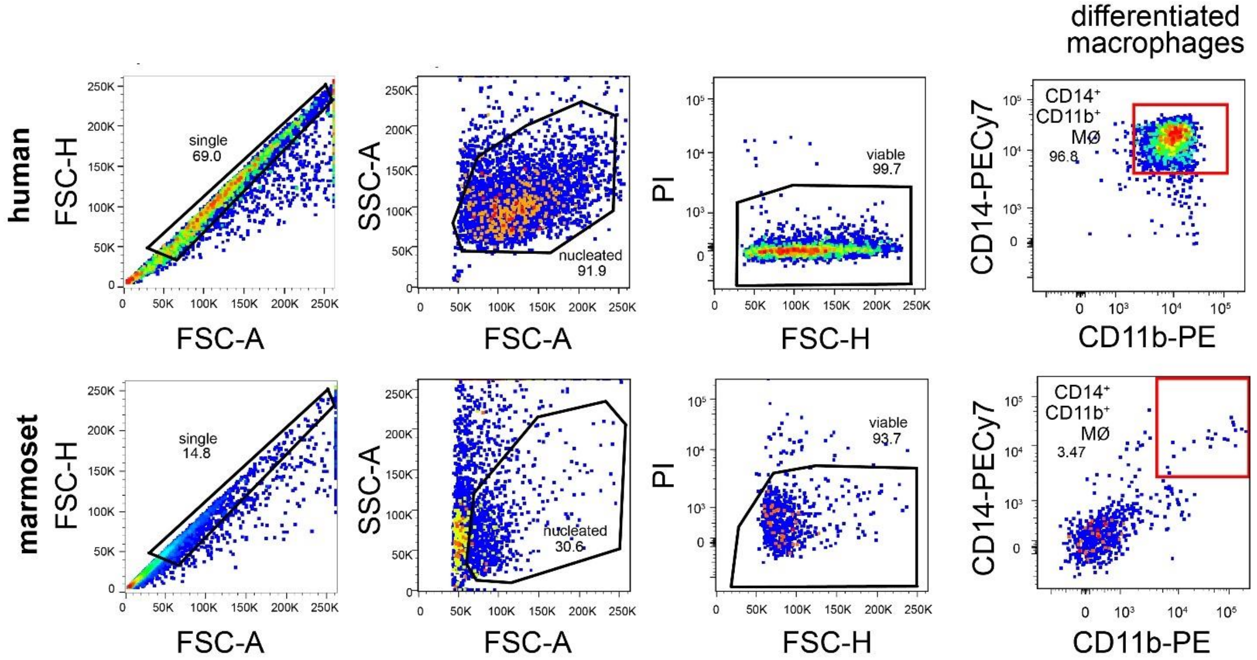

differentiated macrophages
human
marmoset
FSC-H
FSC-A
single
69.0
14.8
SSC-A
nucleated
91.9
30.6
PI
FSC-H
viable
99.7
93.7
CD14-PECy7
CD11b-PE
CD14⁺
CD11b⁺
MØ
96.8
3.47

Supplementary Fig. 7. Flow cytometric gating strategy for analysis of cultured human and marmoset CD14+CD11b+ macrophages derived from peripheral blood monocytes. Expression of LILRB2 on CD14+CD11b+ macrophages is depicted in Figure 4d. FSC: forward scatter; SSC: side scatter; PI: propidium iodide; MØ: macrophages.

Supplementary Table 1. Differentially expressed genes between injured and control astrocyte nuclei, between RTN4- and RTN4+ nuclei, GO analysis and HumanBase Functional Module Detection data. Available for download as a separate xlsx file.

Supplementary Table 2. Key Resource Table.

REAGENT or RESOURCE	SOURCE	IDENTIFIER
Antibodies		
Goat polyclonal anti-NogoA [1:200]	Santa Cruz	Cat#sc-11032; RRID:AB_650319
Rabbit polyclonal anti-NogoA [1:200]	ProSci	Cat#4089; RRID:AB_10905571
Rabbit monoclonal anti-GAP43 [1:500]	Abcam	Cat#ab75810; RRID:AB_1310252
Mouse monoclonal anti-CD44 [1:1000]	CST	Cat#3570; RRID:AB_2076465
Rabbit polyclonal anti-KLF6 [1:500]	LS-Bio	Cat#LS-B1544; RRID:AB_2130434
Rabbit polyclonal anti-LILRB2 [1:1000]	LS-Bio	Cat#LS-B9762; RRID:AB_10966991
Mouse monoclonal anti-LILRB2 (recombinant neutralizing antibody) [1:1000]	R&D Systems	Cat#MAB2078; RRID:AB_2136396
Mouse monoclonal anti-GFAP [1:1000]	Millipore	Cat#MAB360; RRID:AB_11212597
Rabbit polyclonal anti-GFAP [1:1000]	Millipore	Cat#AB5804; RRID:AB_2109645
Rabbit polyclonal anti-Iba1 [1:1000]	Wako	Cat#019-19741; RRID:AB_839504
Goat polyclonal anti-Iba1 [1:1000]	Abcam	Cat#ab5076; RRID:AB_2224402
Rabbit polyclonal anti-TMEM119 [1:1000]	Sigma	Cat#HPA051870; RRID:AB_2681645
Rabbit polyclonal anti-NgR1 [1:500]	Alomone	Cat#ANT-008; RRID:AB_2040180
Rabbit polyclonal anti-S1PR2 [1:1000]	NovusBio	Cat#NBP2-26691
Rabbit polyclonal anti-POSH [1:500]	Sigma	Cat#SAB2103804; RRID:AB_10696343
Rabbit polyclonal anti-Shroom3 [1:500]	ThermoFisher	Cat#PA5-34482; RRID:AB_2551834
Mouse monoclonal anti-RhoA [1:2000]	Abcam	Cat#ab54835; RRID:AB_945224
Rabbit monoclonal anti-ROCK1 [1:500]	CST	Cat#4035; RRID:AB_2238679
Rabbit polyclonal anti-GAPDH [1:5000]	Abcam	Cat#ab9485; RRID:AB_307275
Mouse monoclonal anti-GAPDH [1:5000]	Abcam	Cat#ab9484; RRID:AB_307274
Mouse monoclonal anti-CD51 [1:1000]	Biolegend	Cat#327902; RRID:AB_2129615
Mouse monoclonal anti-CD49d [1:1000]	Biolegend	Cat#304314; RRID:AB_10643278
Mouse monoclonal anti-CD61 [1:500]	BD Biosciences	Cat#348093;

		RRID:AB_400376
Mouse monoclonal anti-CD29 [1:500]	BD Pharmingen	Cat#559883; RRID:AB_398682
Mouse monoclonal anti-CD11b [0.25µg/mL]	BD Biosciences	Cat#347557; RRID:AB_400323
Mouse monoclonal anti-CD14 [0.5µL/ test]	BD Pharmingen	Cat#557742; RRID:AB_396848
Biological Samples		
Marmoset brain tissue	Listed in this table	N/A
Mouse (C57Bl6/J) post-MCAO brain tissue	Gifted from Prof. Christopher G. Sobey	N/A
Human brain tissue	Newcastle Brain Tissue Resource (UK)	N/A
Marmoset cortical astrocytes	Listed in this table	N/A
Mouse cortical astrocytes	Listed in this table	N/A
Marmoset blood	Listed in this table	N/A
Human blood	Australian Red Cross Blood Service, Melbourne	N/A
Chemicals, Peptides, and Recombinant Proteins		
Endothelin-1	Sigma	E7764-
Recombinant rat NogoA Fc Chimera Protein	R&D Systems	2445-NG-050
Recombinant human NogoA Fc Chimera Protein	R&D Systems	3515-NG-050
Mouse monoclonal anti-LILRB2 (recombinant neutralizing antibody) 10ug/mL	R&D Systems	Cat#MAB2078; RRID:AB_2136396
Experimental Models: Cell Lines		
THP-1 monocytic cell line	ATCC	Cat#TIB-202; RRID:CVCL_0006
Normal human astrocytes	Lonza	CC-2565
Marmoset cortical astrocytes	Generated in house	N/A
Mouse (C57Bl6/J) primary cortical astrocytes	Generated in house	N/A
Experimental Models: Organisms/Strains		
Adult common marmosets (*Callithrix Jacchus*)	National Nonhuman Primate Breeding and Research Facility (Monash University)	N/A
Software and Algorithms		
R 4.0.3	CRAN	https://cran.r-project.org/
RStudio v1.4.1103	RStudio, PBC	https://rstudio.com/
Seurat v3.22 & SeuratWrappers	Satija Lab	https://satijalab.org/seurat/
Cellranger v3.1.0	10X Genomics	https://support.10xgenomics.com/single-cell-gene-expression/software/release-notes/build
Scrublet v0.1	Sam Wolock	https://github.com/swolock/scrublet
PANTHER v16.0	University of Southern California	http://www.pantherdb.org/

HumanBase Functional Gene Networks	Flatiron Institute	https://hb.flatironinstitute.org/
ImageJ/ Fiji v2.1.0/1.53c	Rasband	https://imagej.net/Fiji
Photoshop CC 2017	Adobe	https://www.adobe.com/products/photoshop.html
Illustrator CC 2017	Adobe	https://www.adobe.com/products/illustrator.html
Image Studio Lite v5.2.5	LI-COR	https://www.licor.com/bio/image-studio-lite/
Prism v7	GraphPad	https://www.graphpad.com
FlowJo X	FlowJo, LLC	https://www.flowjo.com/solutions/flowjo/downloads
