# Supplementary material for: NogoA-expressing astrocytes limit peripheral macrophage infiltration after ischemic brain injury in primates
Source: Nat Commun. 2021 Nov 25;12:6906. doi: 10.1038/s41467-021-27245-0 (PMC8617297; doi:10.1038/s41467-021-27245-0)
Supplement: Supplementary file 3 — Description of Additional Supplementary Files [file 41467_2021_27245_MOESM3_ESM.docx]

Description of Additional Supplementary Files

Title: Supplementary Data 1

Description: Differentially expressed genes between injured and control astrocyte nuclei, between RTN4- and RTN4+ nuclei, GO analysis and HumanBase Functional Module Detection data.
