# Supplementary material for: NogoA-expressing astrocytes limit peripheral macrophage infiltration after ischemic brain injury in primates
Source: Nat Commun. 2021 Nov 25;12:6906. doi: 10.1038/s41467-021-27245-0 (PMC8617297; doi:10.1038/s41467-021-27245-0)
Supplement: Supplementary file 5 — Reporting Summary [file 41467_2021_27245_MOESM5_ESM.docx]

**Supplementary Information:**


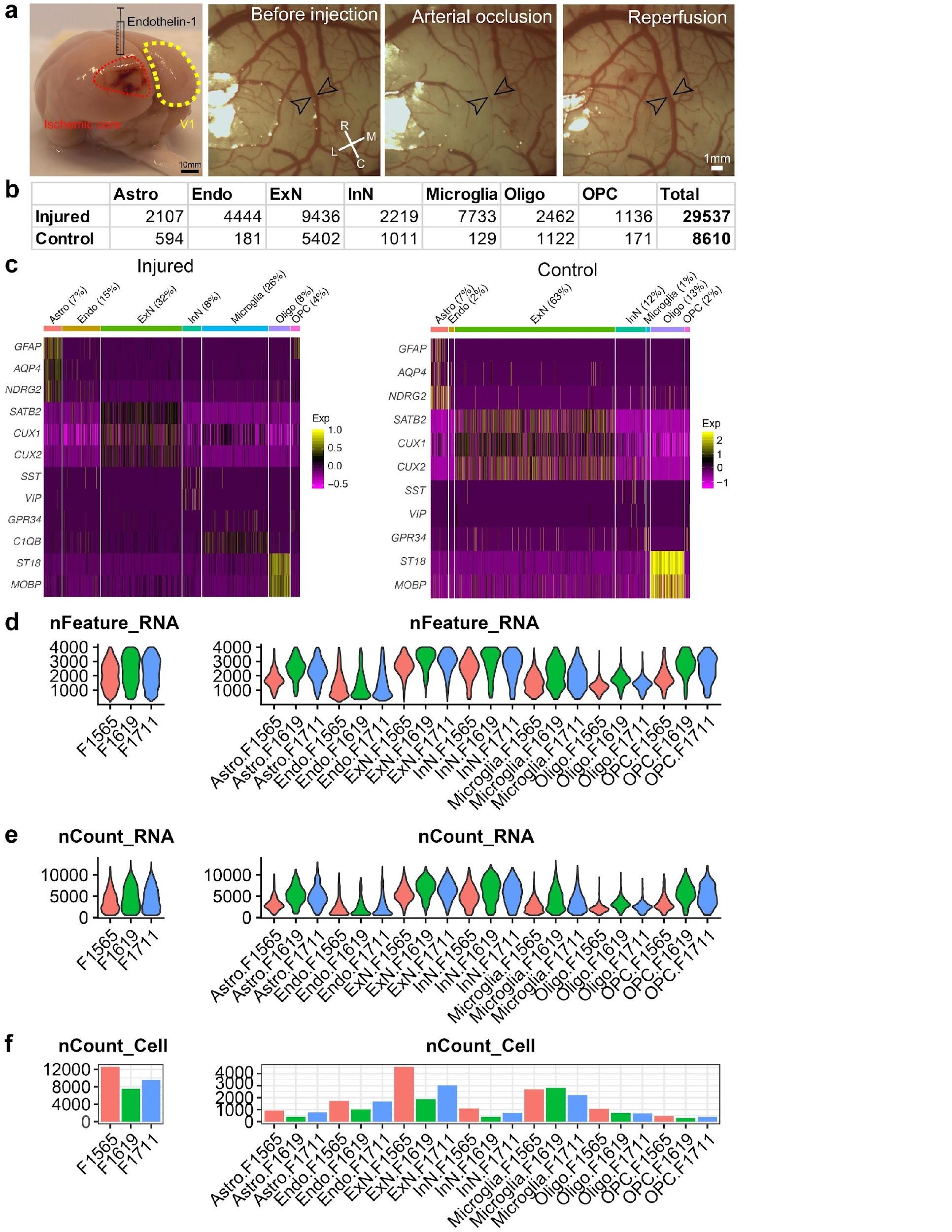


**Supplementary Fig. 1**. Marmoset ischemic stroke induction with vasoconstrictor and astrocyte identification for transcriptomics. (a) Far left; one-week post-endothelin-1-induced ischemia of marmoset caudal neocortex (red hatched area ischemic core and yellow hatched area V1 contralateral, used as uninjured control) and pre- (Middle left)/ post- (Middle & far right images) injection time course highlighting transient ischemia. Arrowheads: occlusion of the posterior cerebral artery. (b) Summary table of single nuclei numbers for each cell type in injured and control cohorts (c) Heat map colored by single nuclei gene expression of remaining cell-type specific markers; accompanies Fig. 1c. (d) Violin plots showing the number of genes detected in each individual injured marmoset (left), and in each cell type from each individual injured marmoset (right). (e) Violin plots showing the distribution of the number of unique molecular identifiers (UMIs) detected in each individual injured marmoset (left), and in each cell type from each individual injured marmoset (right). (f) Bar graphs showing the distribution of the number of cells captured in each individual injured marmoset (left), and in each cell type from each individual injured marmoset (right). Astro: astrocyte; Endo: endothelial cell; ExN: excitatory neurons; InN: inhibitory neurons; Oligo: oligodendrocytes; OPC: oligodendrocyte precursor cells.

**
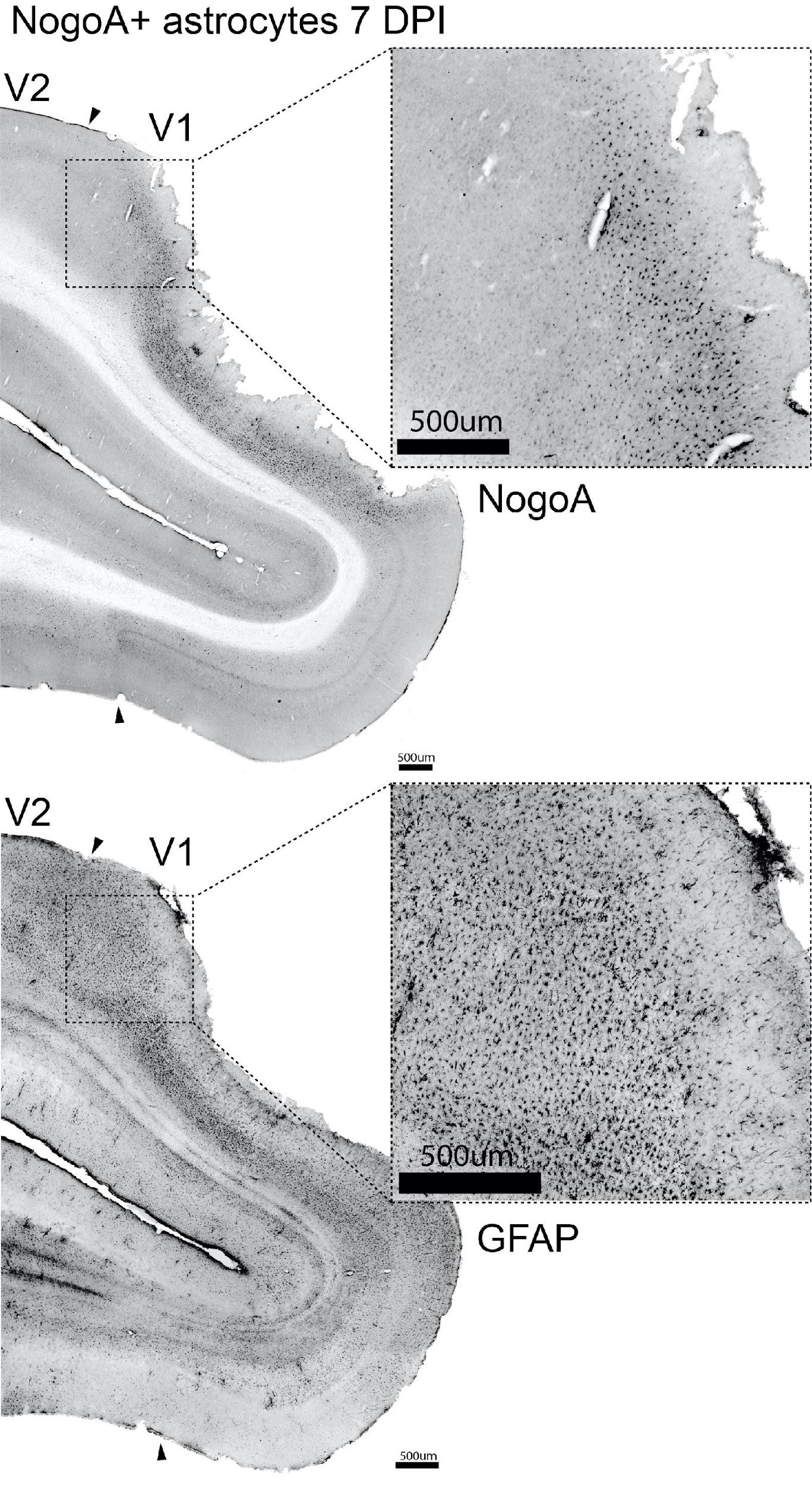
**

**Supplementary Fig. 2**. Sagittal view of NogoA and GFAP expression at the V1/ V2 border post-ischemic stroke. Photomicrographs of marmoset sections 7 DPI (left) and magnified region of interest depicted by black hatched line (right). NogoA (top) and GFAP (bottom) were labelled by DAB immunohistochemistry (n=3 biological replicates). DPI: days post-ischemia; V2: secondary visual area; V1: primary visual cortex; NogoA: neurite outgrowth inhibitor A; GFAP: glial fibrillary acidic protein.

**
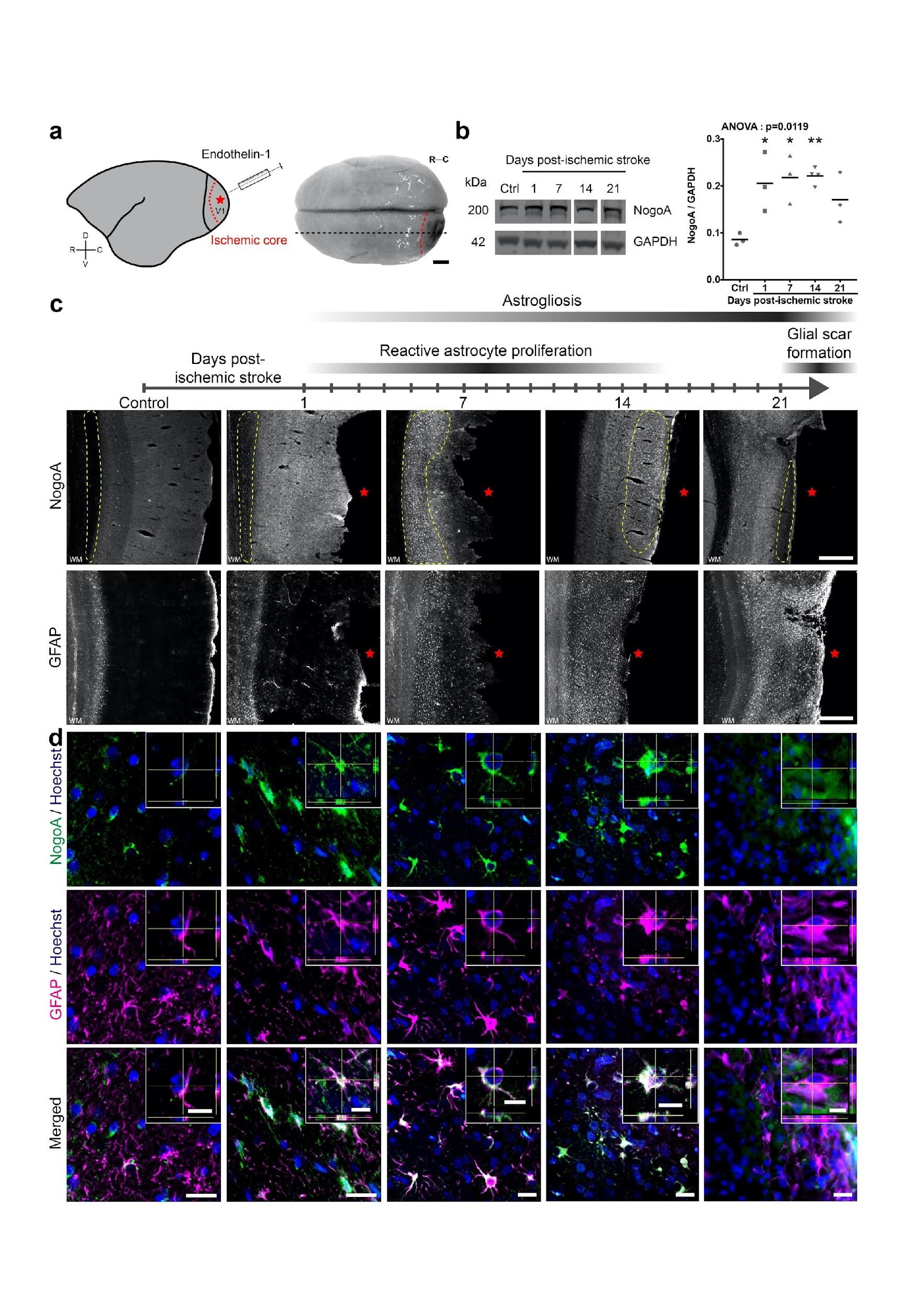
**

**Supplementary Fig. 3**. NogoA is upregulated on marmoset GFAP+ astrocytes post-ischemic stroke. (a) Schematic and image of marmoset brain depicting ischemic-stroke induction, the region of analysis and lesion size relative to brain size. (b) Representative immunoblots for experimental time points analyzed and scatter plots depicting densitometric quantification of NogoA normalized to GAPDH. Each point represents the mean of 3-4 technical replicates for each marmoset biological replicate analyzed, n=3 per time point, excluding 14 DPI where n=4. (c) Schematic of astrocytic pathophysiological time course post-ischemic stroke in primates and representative images showing cellular profile of NogoA and GFAP within marmoset control V1 tissue and ischemic zones at 1, 7, 14 and 21 days post-ischemic stroke (DPI) by DAB immunohistochemistry (n=3 biological replicates/ cohort over several tissue sections comprising the ischemic core). (d) Representative confocal images and stacks with orthogonal views showing NogoA colocalization with GFAP+ cells within yellow dotted line region of interest from (c) in marmoset control V1 tissue and ischemic zones at 1, 7, 14 and 21 DPI (n=3 biological replicates/ cohort over several tissue sections comprising the ischemic core). V1: primary visual cortex; V2: secondary visual area; D: dorsal; V: ventral; R: rostral; C: caudal; kDa: kilodalton; NogoA: neurite outgrowth inhibitor A; GAPDH: glyceraldehyde 3-phosphate dehydrogenase; Ctrl: control; statistical test: ordinary one way-ANOVA with post-hoc Dunnett’s multiple comparisons test; ANOVA p value=0.0119; *: p<0.05; **: p<0.01; WM: white matter; yellow dotted line: cell population of interest; red star: ischemic core; GFAP: glial fibrillary acidic protein; scale bars: 5mm (a), 500μm (c), 20μm (d: single images), 10μm (d: image stacks). Source data and tabulated statistics for (b) are provided as a Source Data file.


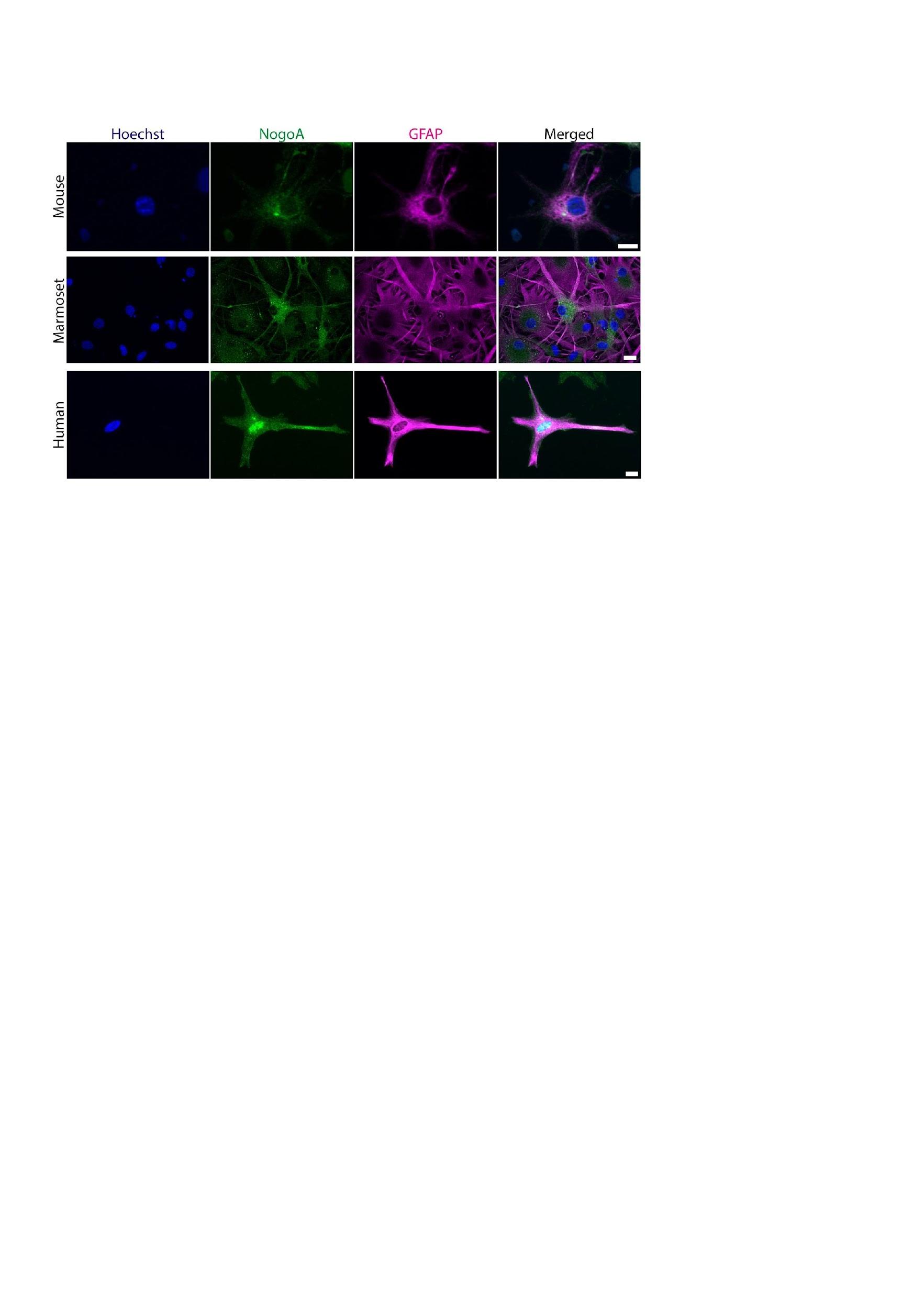


**Supplementary Fig. 4**. Mouse, marmoset and human astrocytes express NogoA *in vitro*, in the absence of myelin. **I**mmunofluorescent images showing expression of NogoA on GFAP+ mouse, marmoset and human astrocytes in culture (n=2 biological replicates/ species). GFAP: glial fibrillary acidic protein; NogoA: neurite outgrowth inhibitor A; scale bars: 20μm.


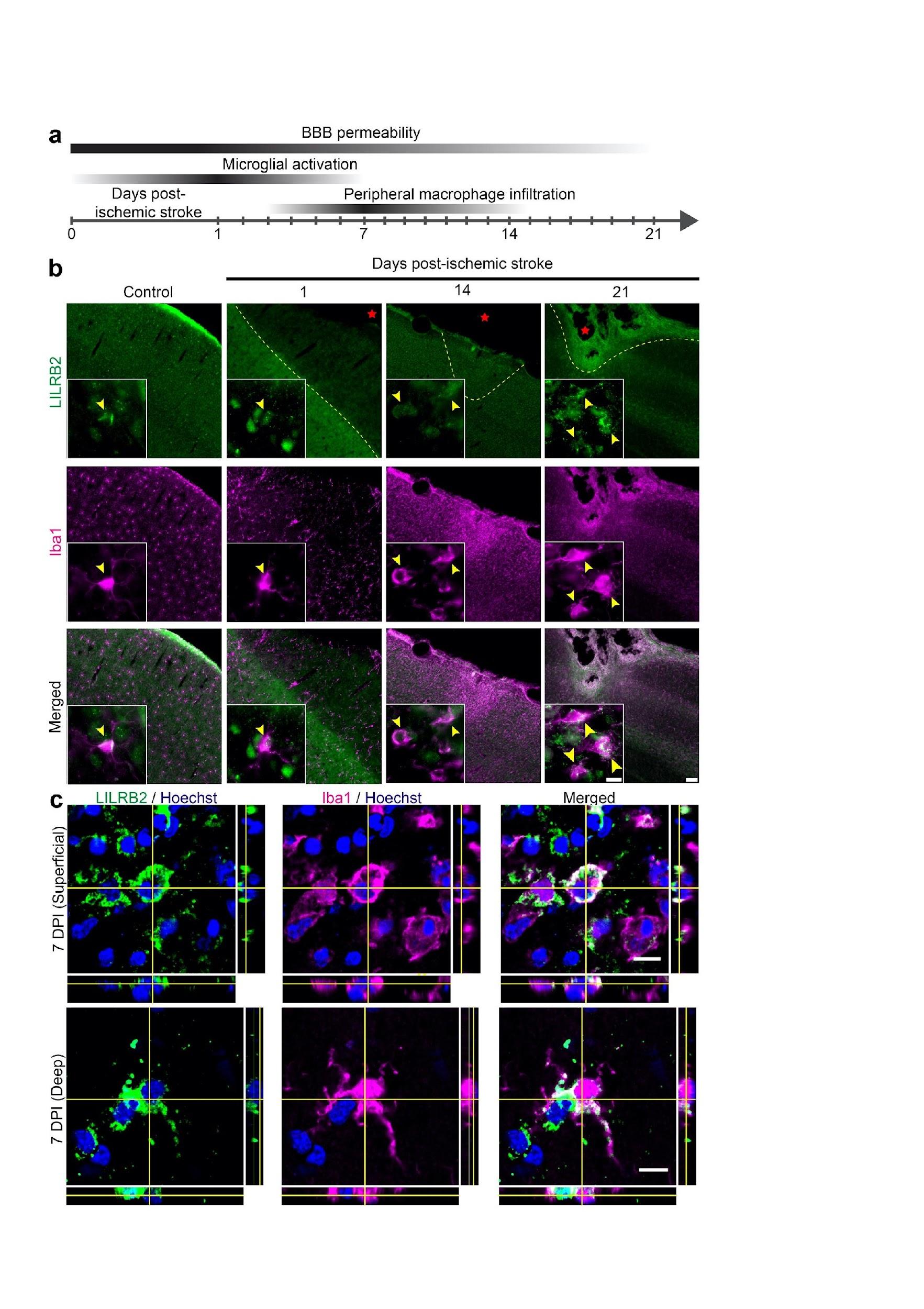


**Supplementary Fig. 5**. LILRB2+/ Iba1+ macrophages at relevant post-ischemic stroke time points. (a) Schematic of macrophagic pathophysiological time course post-ischemic stroke in primates. (b) Representative immunofluorescent images showing LILRB2 colocalization with Iba1+ cells in marmoset control V1 tissue and ischemic zones at 1, 14 and 21 DPI. For 7 DPI time point refer to Fig. 4e. Magnified boxes depict double positive cells and morphologies identified within yellow dotted line region of interest in B (n=3 biological replicates/ cohort over several tissue sections comprising the ischemic core). (c) Confocal image stacks with orthogonal views showing LILRB2 colocalization with Iba1+ cells 7 DPI in superficial and deep cortical tissue (n=3 biological replicates/ cohort over several tissue sections comprising the ischemic core). LILRB2: leukocyte immunoglobulin-like receptor B2; Iba1: ionized calcium-binding adapter molecule 1; yellow dotted line: cell population of interest; red star: ischemic core; yellow arrowheads: LILRB2+/ Iba1+ macrophages; DPI: days post-ischemic stroke; scale bars: 100μm (b: larger box), 10μm (b: magnified box & c).


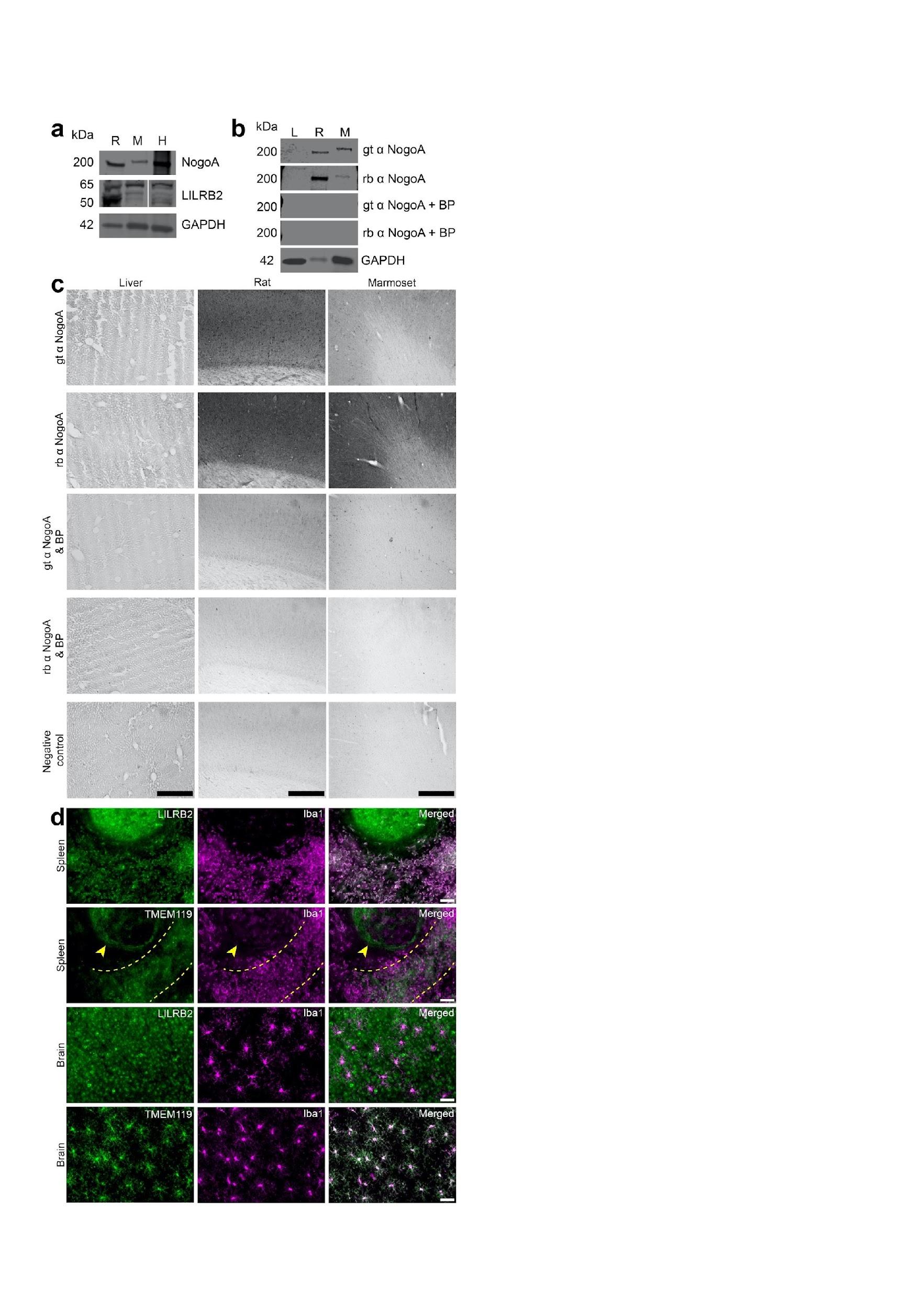


**Supplementary Fig. 6**. NogoA and LILRB2 antibody characterization in rat, marmoset and human. (a) Immunoblots of NogoA and LILRB2 in rat (lane 1), marmoset (lane 2) and human (lane 3) cortical tissue (n=2 biological replicates/ species, each set of samples analyzed across two independent experiments). (b) Immunoblots of two different NogoA antibodies in marmoset liver (lane 1), rat cortical tissue (lane 2) and marmoset cortical tissue (lane 3) (n=1 biological replicate/ tissue, repeated across two independent experiments). (c) Images show specificity of immunostaining by the two NogoA antibodies used in this study in marmoset liver, rat cortical tissue and marmoset cortical tissue (liver: n=1 biological replicate; rat cortical tissue: n=2 biological replicates; marmoset cortical tissue: n>3, over at least 3 tissue sections). (d) Images showing LILRB2 and TMEM119 co-labeled with Iba1 in marmoset spleen and brain (spleen: n=1 biological replicate; brain: n>3 biological replicates, over at least 3 tissue sections). NogoA: neurite outgrowth inhibitor A; LILRB2: leukocyte immunoglobulin-like receptor B2; GAPDH: glyceraldehyde 3-phosphate dehydrogenase; kDa: kilodalton; gt: goat; rb: rabbit; α: anti-; BP: blocking peptide; TMEM119: transmembrane protein 119; Iba1: ionized calcium-binding adapter molecule 1; yellow dotted line: reticular fibers of splenic cord; yellow arrowhead: lymph node capsule; scale bars: 500µm (c), 50µm (d). Source data are provided as a Source Data file.


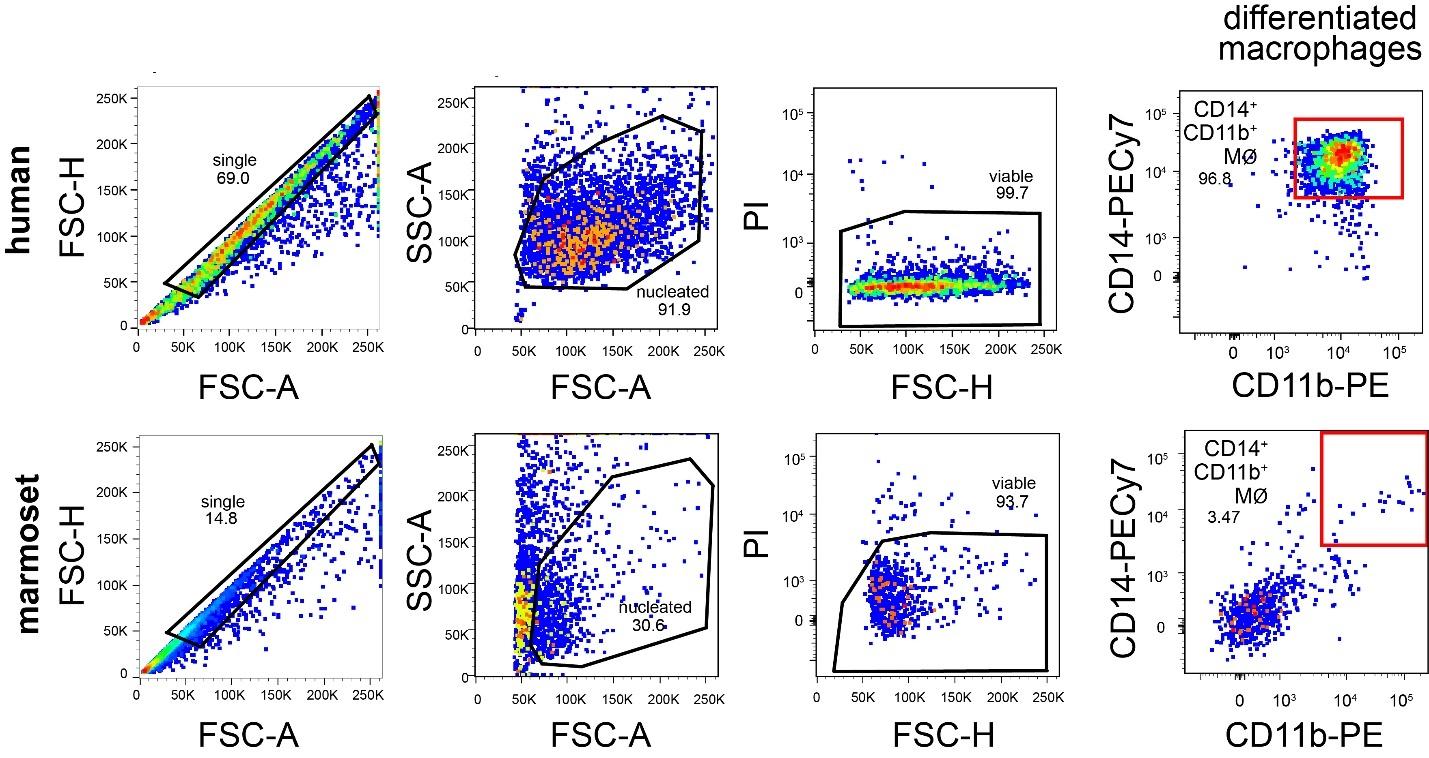


**Supplementary Fig. 7**. Flow cytometric gating strategy for analysis of cultured human and marmoset CD14+CD11b+ macrophages derived from peripheral blood monocytes. Expression of LILRB2 on CD14+CD11b+ macrophages is depicted in Figure 4d. FSC: forward scatter; SSC: side scatter; PI: propidium iodide; MØ: macrophages.

**Supplementary Table 1.** Key Resource Table.

| REAGENT or RESOURCE | SOURCE | IDENTIFIER |
| --- | --- | --- |
| Antibodies | | |
| Goat polyclonal anti-NogoA [1:200] | Santa Cruz | Cat#sc-11032; RRID:AB_650319 |
| Rabbit polyclonal anti-NogoA [1:200] | ProSci | Cat#4089; RRID:AB_10905571 |
| Rabbit monoclonal anti-GAP43 [1:500] | Abcam | Cat#ab75810; RRID:AB_1310252 |
| Mouse monoclonal anti-CD44 [1:1000] | CST | Cat#3570; RRID:AB_2076465 |
| Rabbit polyclonal anti-KLF6 [1:500] | LS-Bio | Cat#LS-B1544; RRID:AB_2130434 |
| Rabbit polyclonal anti-LILRB2 [1:1000] | LS-Bio | Cat#LS-B9762; RRID:AB_10966991 |
| Mouse monoclonal anti-LILRB2 (recombinant neutralizing antibody) [1:1000] | R&D Systems | Cat#MAB2078; RRID:AB_2136396 |
| Mouse monoclonal anti-GFAP [1:1000] | Millipore | Cat#MAB360; RRID:AB_11212597 |
| Rabbit polyclonal anti-GFAP [1:1000] | Millipore | Cat#AB5804; RRID:AB_2109645 |
| Rabbit polyclonal anti-Iba1 [1:1000] | Wako | Cat#019-19741; RRID:AB_839504 |
| Goat polyclonal anti-Iba1 [1:1000] | Abcam | Cat#ab5076; RRID:AB_2224402 |
| Rabbit polyclonal anti-TMEM119 [1:1000] | Sigma | Cat#HPA051870; RRID:AB_2681645 |
| Rabbit polyclonal anti-NgR1 [1:500] | Alomone | Cat#ANT-008; RRID:AB_2040180 |
| Rabbit polyclonal anti-S1PR2 [1:1000] | NovusBio | Cat#NBP2-26691 |
| Rabbit polyclonal anti-POSH [1:500] | Sigma | Cat#SAB2103804; RRID:AB_10696343 |
| Rabbit polyclonal anti-Shroom3 [1:500] | ThermoFisher | Cat#PA5-34482; RRID:AB_2551834 |
| Mouse monoclonal anti-RhoA [1:2000] | Abcam | Cat#ab54835; RRID:AB_945224 |
| Rabbit monoclonal anti-ROCK1 [1:500] | CST | Cat#4035; RRID:AB_2238679 |
| Rabbit polyclonal anti-GAPDH [1:5000] | Abcam | Cat#ab9485; RRID:AB_307275 |
| Mouse monoclonal anti-GAPDH [1:5000] | Abcam | Cat#ab9484; RRID:AB_307274 |
| Mouse monoclonal anti-CD51 [1:1000] | Biolegend | Cat#327902;  RRID:AB_2129615 |
| Mouse monoclonal anti-CD49d [1:1000] | Biolegend | Cat#304314;  RRID:AB_10643278 |
| Mouse monoclonal anti-CD61 [1:500] | BD Biosciences | Cat#348093;  RRID:AB_400376 |
| Mouse monoclonal anti-CD29 [1:500] | BD Pharmingen | Cat#559883;  RRID:AB_398682 |
| Mouse monoclonal anti-CD11b [0.25µg/mL] | BD Biosciences | Cat#347557;  RRID:AB_400323 |
| Mouse monoclonal anti-CD14 [0.5µL/ test] | BD Pharmingen | Cat#557742;  RRID:AB_396848 |
| Biological Samples | | |
| Marmoset brain tissue | Listed in this table | N/A |
| Mouse (C57Bl6/J) post-MCAO brain tissue | Gifted from Prof. Christopher G. Sobey | N/A |
| Human brain tissue | Newcastle Brain Tissue Resource (UK) | N/A |
| Marmoset cortical astrocytes | Listed in this table | N/A |
| Mouse cortical astrocytes | Listed in this table | N/A |
| Marmoset blood | Listed in this table | N/A |
| Human blood | Australian Red Cross Blood Service, Melbourne | N/A |
| Chemicals, Peptides, and Recombinant Proteins | | |
| Endothelin-1 | Sigma | E7764- |
| Recombinant rat NogoA Fc Chimera Protein | R&D Systems | 2445-NG-050 |
| Recombinant human NogoA Fc Chimera Protein | R&D Systems | 3515-NG-050 |
| Mouse monoclonal anti-LILRB2 (recombinant neutralizing antibody) 10ug/mL | R&D Systems | Cat#MAB2078; RRID:AB_2136396 |
| Experimental Models: Cell Lines | | |
| THP-1 monocytic cell line | ATCC | Cat#TIB-202; RRID:CVCL_0006 |
| Normal human astrocytes | Lonza | CC-2565 |
| Marmoset cortical astrocytes | Generated in house | N/A |
| Mouse (C57Bl6/J) primary cortical astrocytes | Generated in house | N/A |
| Experimental Models: Organisms/Strains | | |
| Adult common marmosets (*Callithrix Jacchus*) | National Nonhuman Primate Breeding and Research Facility (Monash University) | N/A |
| Software and Algorithms | | |
| R 4.0.3 | CRAN | https://cran.r-project.org/ |
| RStudio v1.4.1103 | RStudio, PBC | https://rstudio.com/ |
| Seurat v3.22 & SeuratWrappers | Satija Lab | https://satijalab.org/seurat/ |
| Cellranger v3.1.0 | 10X Genomics | https://support.10xgenomics.com/single-cell-gene-expression/software/release-notes/build |
| Scrublet v0.1 | Sam Wolock | https://github.com/swolock/scrublet |
| PANTHER v16.0 | University of Southern California | http://www.pantherdb.org/ |
| HumanBase Functional Gene Networks | Flatiron Institute | https://hb.flatironinstitute.org/ |
| ImageJ/ Fiji v2.1.0/1.53c | Rasband | https://imagej.net/Fiji |
| Photoshop CC 2017 | Adobe | https://www.adobe.com/products/ photoshop.html |
| Illustrator CC 2017 | Adobe | https://www.adobe.com/products/ illustrator.html |
| Image Studio Lite v5.2.5 | LI-COR | https://www.licor.com/bio/image-studio-lite/ |
| Prism v7 | GraphPad | https://www.graphpad.com |
| FlowJo X | FlowJo, LLC | https://www.flowjo.com/solutions/flowjo/downloads |
